# Transcriptome changes in response to temperature in the fish pathogen *Photobacterium damselae* subsp. *damselae*: Clues to understand the emergence of disease outbreaks at increased seawater temperatures

**DOI:** 10.1371/journal.pone.0210118

**Published:** 2018-12-31

**Authors:** Xosé M. Matanza, Carlos R. Osorio

**Affiliations:** Departamento de Microbioloxía e Parasitoloxía, Instituto de Acuicultura, Universidade de Santiago de Compostela, Santiago de Compostela, Spain; Universidade de Coimbra, PORTUGAL

## Abstract

The marine bacterium *Photobacterium damselae* subsp. *damselae* (*Pdd*) is a generalist and facultative pathogen that causes disease in a wide range of marine animals including fish species of importance in aquaculture. Disease outbreaks in fish farms have been correlated with an increased water temperature during summer months. In this study, we have used RNA sequencing to analyze the transcriptome of *Pdd* RM-71 cultured at two different temperatures, which simulated temperature conditions experienced during free swimming lifestyle at mid latitudes in winter months (15°C) and during outbreaks in aquaculture in warm summer months (25°C). The enhanced bacterial growth of *Pdd* observed at 25°C in comparison to 15°C suggests that an elevated seawater temperature contributes to the build-up of a sufficient bacterial population to cause disease. In comparison to growth at 15°C, growth at 25°C resulted in the upregulation of genes involved in DNA synthesis, nutrient uptake, chemotaxis, flagellar motility, secretion systems and antimicrobial resistance. Plasmid-encoded virulence factors, which include a putative adhesin/invasin OmpU, a transferrin receptor and a serum resistance protein, were also upregulated. Transcription factor RpoS, genes involved in cold shock response, modulation of cell envelope and amino acid metabolism, as well as genes of yet unknown function were downregulated at 25°C. Notably, the gene encoding damselysin cytotoxin (Dly) was among the most highly transcribed genes at the two assayed temperatures, at levels comparable to the most highly expressed housekeeping genes. This study contributes to our understanding of the regulatory networks and biology of a generalist marine bacterial pathogen, and provides evidence that temperature regulates multiple physiological and virulence-related functions in *Pdd*.

## Introduction

The *Vibrionaceae* family includes aquatic bacteria found in ocean environments and temperature and salinity are the main abiotic factors that shape their abundance and distribution [1, 2]. Rising ocean temperatures contribute to the increase in prevalence and severity of a wide range of Vibrio-related diseases in marine organisms and also in humans [3, 4]. *Vibrio* and *Photobacterium* infections have a marked seasonal distribution, and most cases occur during the warmer summer months [5].

*Photobacterium damselae* subsp. *damselae* (hereafter *Pdd*), a marine bacterium of the *Vibrionaceae* family, is a pathogen of a wide range of marine animals, including fish, molluscs, crustaceans and cetaceans. It is also an emerging pathogen in marine aquaculture systems that causes wound infections and septicaemia in economically important fish species [6–8]. In addition, it is an opportunistic human pathogen which causes severe wound infections that can have a fatal outcome [9].

Notably, disease outbreaks in aquaculture caused by *Pdd* have been associated with an increased seawater temperature during summer months. This has been the case for outbreaks in farms rearing turbot (*Scophthalmus maximus*) [10], rainbow trout (*Oncorhynchus mykiss*) [11, 12], seabass (*Dicentrarchus labrax*) [13–16], gilthead seabream (*Sparus aurata*) [14, 15, 17], cobia (*Rachycentron canadum*) [18] and silver pomfret (*Pampus argenteus*) [19]. There is increasing evidence that outbreaks of *Pdd* in fish farms are caused by genetically heterogeneous populations existing in the environment [7, 20]. It is believed that under advantageous environmental conditions these populations cause disease by taking advantage of stressed fish hosts.

The major reported virulence factors of *Pdd* are cytotoxins with hemolytic activity [8]. Highly virulent strains harbor the virulence plasmid pPHDD1 that carry the cytotoxin genes *dly* and *hlyA*_*pl*_ [21]. *dly* encodes damselysin toxin (Dly), a phospholipase-D active against sphingomyelin [22], and *hlyA*_*pl*_ encodes the pore-forming toxin phobalysin P (PhlyP) [23]. In addition, the pore-forming toxin phobalysin C (PhlyC), encoded by the *hlyA*_*ch*_ gene located on chromosome I, and the phospholipase PlpV also contribute to virulence in fish and to cell toxicity [24, 25]. These four cytotoxins are secreted via a type II secretion system [26]. The two-component regulatory system RstAB positively regulates transcription of the hemolysin genes *dly*, *hlyA*_*pl*_ and *hlyA*_*ch*_, and its inactivation severely impairs virulence [27].

Despite the increasing evidence suggesting that outbreaks caused by *Pdd* in fish farms are triggered by rises in seawater temperature during summer months, the role of temperature in the physiology and gene regulation of this pathogen has not been studied so far. The aim of this study was to investigate the transcriptome of *Pdd* at two different temperatures, 15°C and 25°C, using an RNA-seq approach. We identified a number of genes upregulated at 25°C—the temperature at which most outbreaks occur in aquaculture farms—that likely contribute to an increased bacterial growth rate and to an enhanced ability to colonize and survive in fish hosts. Additionally, we found genes with higher expression at 15°C which might aid *Pdd* to adapt to life in colder waters during winter months. The global transcriptome data also shed light on the relative expression values of genes encoding virulence factors compared to housekeeping genes and demonstrated that the damselysin toxin gene is one of the most highly expressed genes in the cell. Finally, this study has brought to the forefront many previously overlooked genetic networks and gene clusters of this pathogen. Overall, the information generated in this study is expected to provide novel approaches for the prevention and control of vibriosis caused by *Pdd* in marine fish aquaculture.

## Materials and methods

### Growth analysis

Cells were routinely grown at 15 or 25°C on tryptic soy agar (TSA) or in tryptic soy broth (TSB) supplemented with NaCl up to 1% (TSA-1 and TSB-1, respectively). For growth curves, three replicates for each temperature of the assay (15 and 25°C) were grown in TSB-1 until obtaining exponentially growing precultures (OD_600_: 0.3). Then, 1:100 dilutions of each preculture were grown in 100 μl of TSB-1 in 96 well plates and the optical density (OD_600_) was measured during 48h using the spectrophotometer Epoch2 microplate reader (Biotek). Three replicates were measured for each temperature condition.

### RNA-seq

#### Experimental design, RNA extraction and purification

As for RNA-seq approach, 3 biological replicates were performed for each condition. 15°C was chosen as the control condition and is close to the temperature that this bacterium finds during free swimming lifestyle in mid latitudes, whilst 25°C represents the higher temperature condition that usually precedes aquaculture outbreaks. For each temperature, three independent precultures were started and grown until an OD_600_: 0.3. Then, 1:100 dilutions of each preculture were grown in 10 ml of TSB-1 in 100 ml flasks until they reached a sharp OD_600_ of 0.55. Cells were immediately treated with RNAprotect Bacteria Reagent (Qiagen) for stabilization of RNA following manufacturer’s instructions. Pelleted cells were then carefully resuspended in TE buffer (30mM Tris·Cl, 1mM EDTA, pH 8.0) containing 15mg/ml lysozyme (Sigma Aldrich) and the appropriate volume of Proteinase K (Qiagen). RNA extraction was subsequently carried out using RNeasy Mini Kit (Qiagen) following manufacturers’ instructions. An extra DNase I treatment was carried out using the on-column kit RNase-free DNase (Qiagen) to eliminate genomic DNA contamination. RNA was eluted using nuclease-free water. The quality and the quantity of the total RNA was determined using a Bioanalyzer 2100 (RNA 6000 Nano chip assay) and a Qubit 3.0 (Quant-It dsRNA BR Assay).

#### Libraries preparation and sequencing

Total RNA was rRNA-depleted using the Ribo-Zero rRNA Removal Kit (Gram Negative Bacteria) (Illumina) and cDNA libraries were obtained using the TruSeq RNA kit following Illumina´s recommendations. Briefly, rRNA-depleted RNA was chemically fragmented prior to reverse transcription and cDNA generation. The cDNA fragments then went through an end repair process, the addition of a single ‘A’ base to the 3’ end and then ligation of the adapters. Finally, the products were purified and enriched by PCR to create the indexed final double stranded cDNA library. The pool of libraries was sequenced on an Illumina HiSeq 2500 sequencer.

#### Mapping and quantification of transcripts

The quality control of the raw data (raw reads) was performed using the FastQC [http://www.bioinformatics.babraham.ac.uk/projects/fastqc/] program. The raw pair-end reads were first mapped against the reference genome of the *Photobacterium damselae* subsp. *damselae* type strain CIP102761 (GenBank Acc. No. NZ_ADBS00000000.1). Reads that did not map to the reference genome (corresponding to genes of RM-71 strain not present in CIP102761) were subsequently mapped to the draft genome sequence of strain RM-71 (GenBank Acc. No. NZ_LYBT00000000.1). The two processes were completed using the Bowtie2 [28] v2.2.6 algorithm. Several quality control steps were performed. Reads displaying a very low quality were removed by using Samtools [29] and Picard Tools software [30]. Furthermore, one of the key factors that can condition the sequencing process is the GC content of samples which was checked as normal (distribution between 40–60%) in our experiment. Likewise, distribution of duplicates was evaluated to confirm the normal small proportion. The process of genetic quantification was carried out by the HTSeq [31] software (0.6.1 version).

#### Comparison between samples

Concordance between samples of the same condition (replicates of each of the two assayed temperatures) was carried out by a study of correlation and distance considering the whole transcriptome normalized by the size of the library. This process was made using the statistics program R. Differential expression analysis was assessed using DESeq2 [32] method (1.18.1 version). The analysis of Differentially Expressed Genes (DEG) was done by using statistical packages designed by Python and R, using the DESseq2 [32] algorithm applying a differential negative binomial distribution for the statistics significance. Comparison between the two different conditions (25°C vs. 15°C) was set as fixed effect in DEseq2. A Python script developed at Sistemas Genómicos (Valencia, Spain) was employed to generate a data matrix for each group condition with the counts obtained from HTSeq count for each sample (each of the three replicates at each of the two temperatures). We considered differentially expressed genes those with Fold Change (FC) value lower than -1.5 or higher than 1.5 and a P value adjusted by False Discovery Rate (FDR) [33] ≤0.05. FPKM (Fragments per kilobase per million fragments mapped) values calculated with Cufflinks v2.2.1 [34] were used to represent the expression of each individual gene. FPKM is used for normalization of the data since it indicates the number of lectures of a given gene per kilobase (independently of the length of the gene), and per million reads (independently of the size of the library).

## Results and discussion

### Growth dynamics of *Pdd* at 15 and 25°C

**Fig 1:**
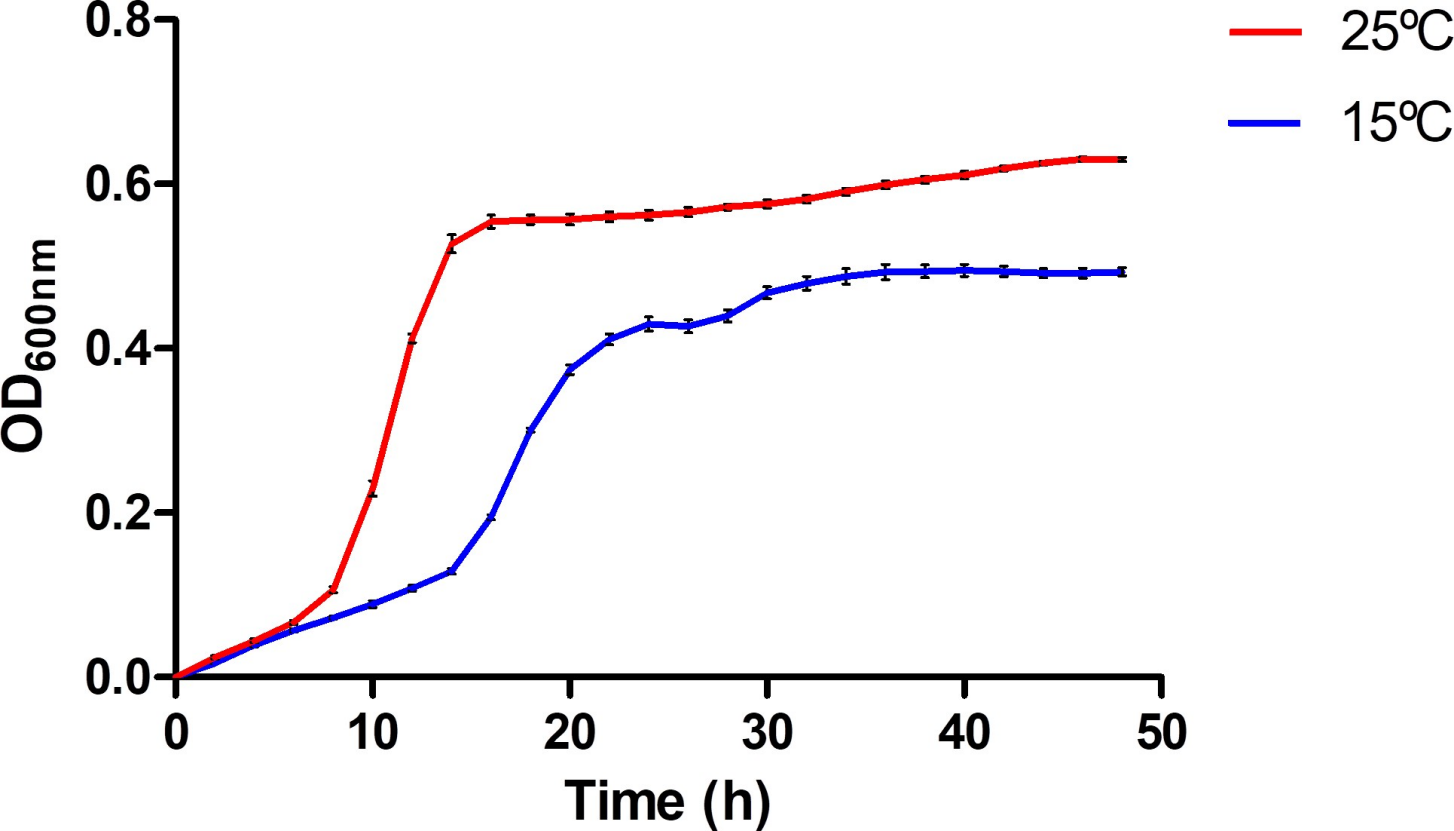
The influence of temperature cultivation in growth dynamics of *Pdd*. Growth of RM-71 strain was assayed at 15°C and 25°C in TSB-1 for 48 h. Vertical error bars represent standard deviation of biological triplicates.

*Pdd* RM-71 was the strain selected for the present study. It was isolated during a disease outbreak in a turbot (*Scophthalmus maximus*) farm in Galicia (NW Spain), when the water temperature increased suddenly from 18°C to 22–24°C in the summer of 1988 [10]. It is a highly virulent, strongly hemolytic and cytotoxic strain and contains the pPHDD1 virulence plasmid [21]. Growth of RM-71 was analyzed in 48-h continuous cultures at 15 and 25°C in TSB-1 medium (S1 Table). The two assayed temperatures simulated an *a priori* non-risky condition (15°C) and warm water episodes that trigger aquaculture outbreaks (25°C). The beginning of the exponential phase was delayed at 15°C compared to growth at 25°C and there was a great difference between optical density values after 15 h of cultivation at 15°C (OD_600_: 0.129) and 25°C (OD_600_: 0.527) (Fig 1). These observations suggest that 25°C is closer to the optimal growth temperature of *Pdd* than 15°C.

This substantial increase in bacterial proliferation at 25°C with respect to 15°C during the first 15 h of cultivation might contribute to the rapid progression of *Pdd* outbreaks when the sea water temperature increases. Proliferation of species of the *Vibrionaceae* family is favoured by warm (>15°C) sea waters [35], and recent studies have demonstrated that following an increase in water temperature, Vibrios can go from barely detectable to being the predominant bacteria in a very short time [36, 37]. *Pdd* isolation was first reported from ulcers in damselfish (*Chromis punctipinnis*) during the summer and fall seasons in southern California and was shown to follow a seasonal pattern of infectivity. It was proposed that elevated water temperatures might allow the build-up of sufficient bacterial populations to cause disease in damselfish, hence the seasonal infectivity of *Pdd* [38]. Previous studies have provided sound evidence that seawater transmits the disease caused by *Pdd* and that the spread of this bacterium largely depends on water temperature [39]. Skin is suggested to be a potential route of penetration for this pathogen, which is able to specifically adhere to fish mucus [39]. Hence, even if fish are colonized by a small number of bacteria, fast proliferation enhanced by warm temperature will facilitate the infecting bacterial population to evade host immune responses by a variety of mechanisms [40]. High numbers of bacterial cells at the infection site might cause exhaustion of complement components as well as of phagocytes [41] leading to systemic infection and fish death. Overall, the results of the growth dynamics analysis at 15 and 25°C contribute to understand why increased water temperatures precede most outbreaks caused by this pathogen.

### RNA sequencing results

**Fig 2:**
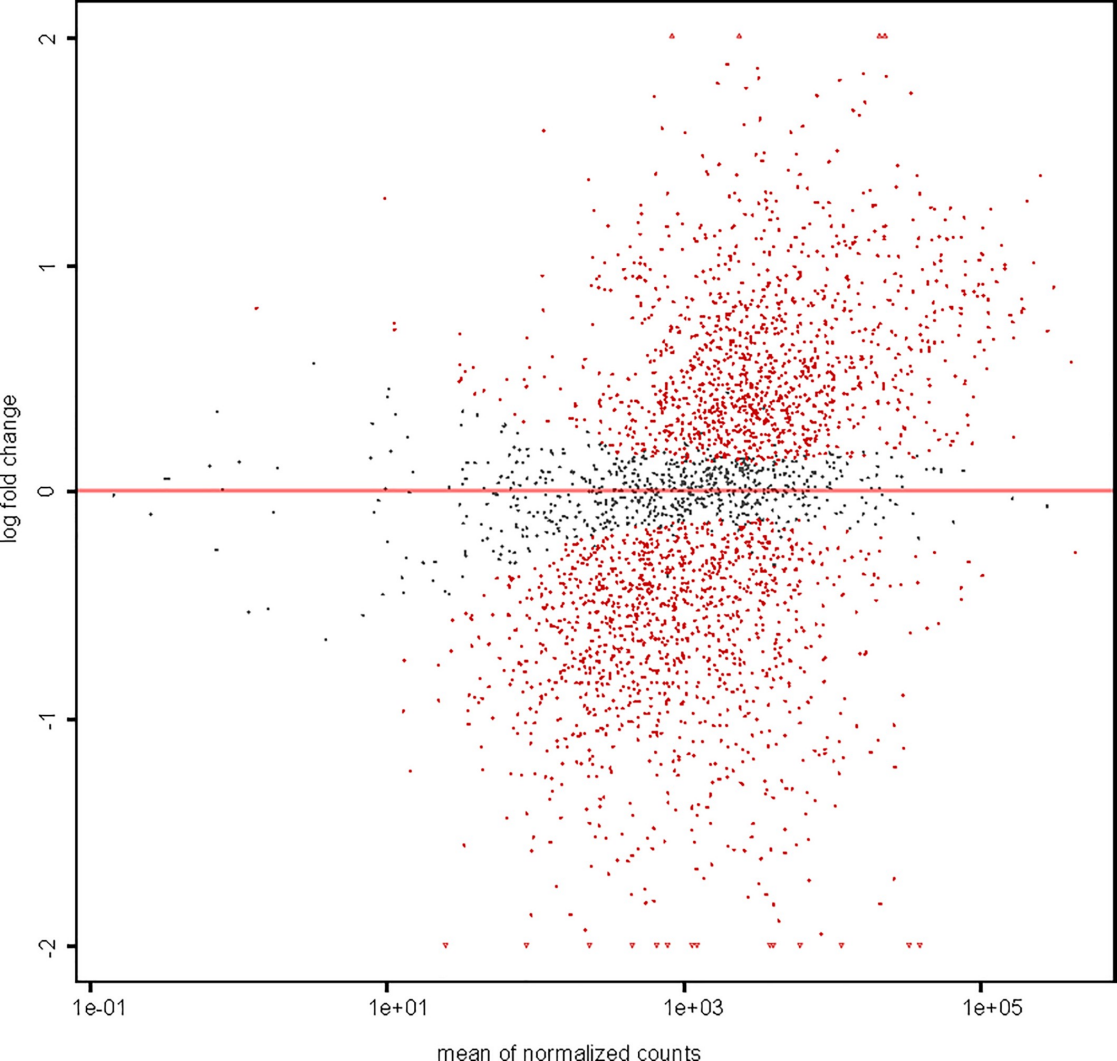
Smear plot of differentially expressed genes (DEGs) in *Pdd* RM-71 exposed to two temperatures, 15°C and 25°C. The smear plot shows the relationship between the log FC and mean of normalized counts. Grey points represent genes with non-significant changes in expression, whereas red points represent genes that are significantly differentially expressed.

Strain RM-71 was grown at 15 and 25°C, and cDNA prepared from mRNA isolated from cultures at the two different temperatures was subjected to Illumina sequencing. Around 55 to 71 million reads were generated for each biological replicate (S2 Table). Growth at 15°C was defined as the control condition. The comparative analyses of the transcriptomes at 15 and 25°C resulted in a total of 1195 differentially expressed genes (DEGs): 641 genes with lower expression at 25°C (FC lower than -1.5) and 554 genes with higher expression at 25°C (FC higher than 1.5) (Fig 2, S3 and S4 Tables).

**Fig 3:**
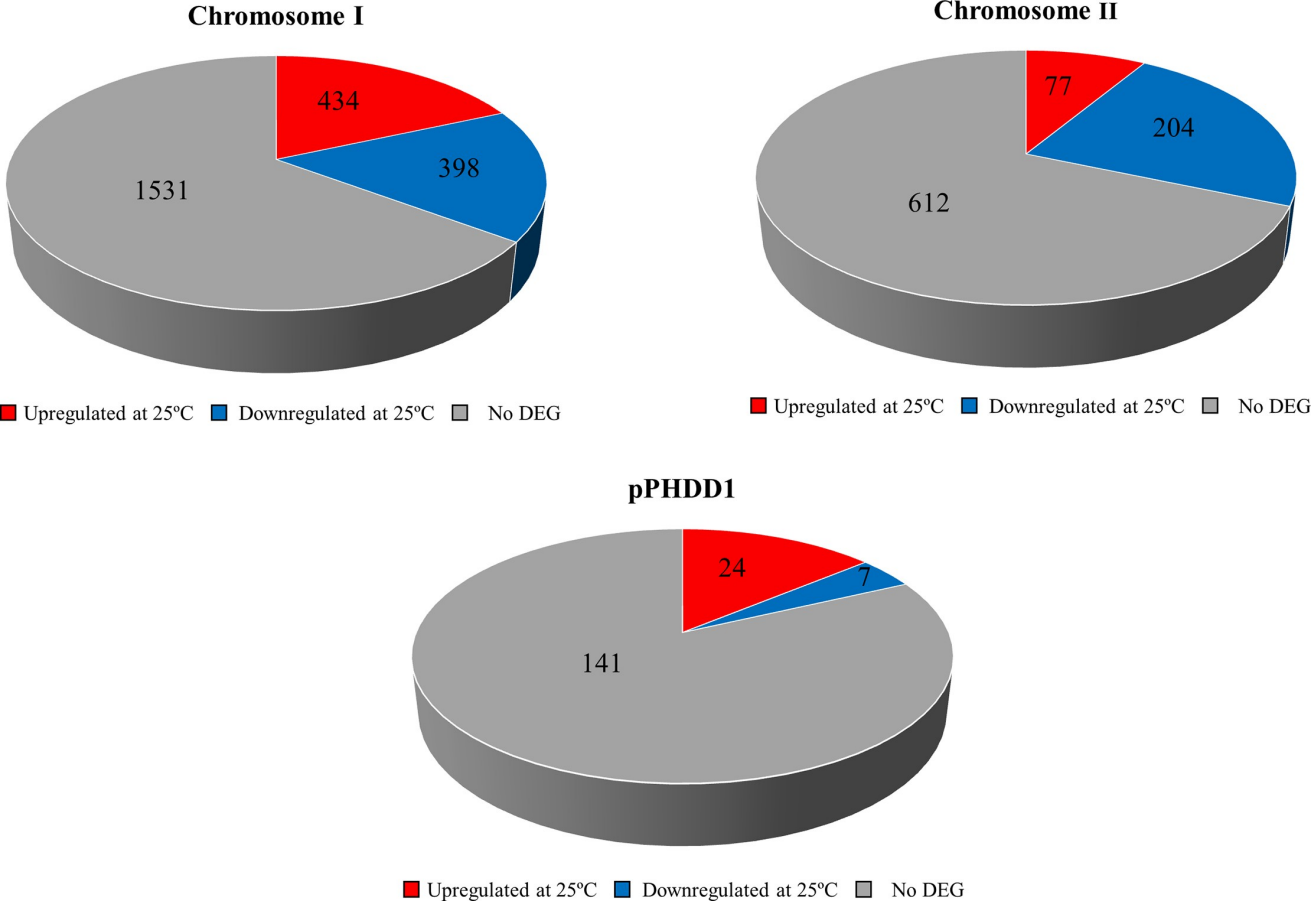
Graphical representation of DEGs distribution among the two *Pdd* chromosomes and pPHDD1 virulence plasmid. Numbers denote DEGs upregulated at 25°C (red), downregulated at 25°C (blue), and genes not differentially regulated (grey).

Similar to other members of the *Vibrionaceae* family, *Pdd* RM-71 contains two chromosomes. In addition, this strain harbors the virulence plasmid pPHDD1 [21]. Using the complete sequences of chromosome I and II of the type strain CIP102761, and the complete pPHDD1 plasmid sequence of strain RM-71 (GenBank Acc. No. NC_014653) as references, we distributed the DEGs into each replicon. Notably, we observed an imbalance in the number of DEGs between the two chromosomes (Fig 3). In chromosome I similar numbers of DEGs are upregulated and downregulated. However, chromosome II contains 204 downregulated genes and only 77 upregulated genes at 25°C. The chromosome I of *Vibrionaceae* species contains most of the essential genes, whereas chromosome II has a more flexible gene content and is responsible for adaptation to environmental changes [42, 43]. Interestingly, among the 31 DEGs in the virulence plasmid pPHDD1, 24 corresponded to genes whose expression is upregulated at 25°C, an observation of special interest since this plasmid constitutes a hallmark of highly virulent isolates [8]. Indeed, the present study has unveiled potential virulence factors encoded by pPHDD1 plasmid among the group of genes upregulated at 25°C (see below), thus highlighting the importance of this virulence plasmid in the pathobiology of *Pdd*.

### Genes involved in growth and virulence are upregulated at 25°C

Growth at 25°C resulted in the upregulation of 533 genes that mapped to the genome of the type strain (S3 Table) and of 21 additional genes unique to strain RM-71 (S4 Table). A list of the 50 top DEGs upregulated at 25°C plus additional selected genes is shown in Table 1.

**Table 1:** List of selected DEGs with enhanced expression at 25°C including the 50 top upregulated genes at 25°C. Enhanced expression at 25°C is denoted by positive FC values. Genes with VDA codes correspond to the annotation in the CIP102761 genome, and genes with A0J47 codes correspond to the annotation in the RM-71 genome.

Gene ID	Product/Function	Fold Change	p-value	Location
*Nutrient acquisition/metabolism*				
VDA_001789	Nucleoside permease NupC	5.1	2.2182E-122	ChrI
VDA_001833	GPR1/FUN34/yaaH putative acetate transporter	3.6	5.07132E-17	ChrI
VDA_002532	Porin	5.1	1.08929E-77	ChrI
VDA_003254	Porin	3.0	3.53973E-49	ChrI
VDA_001005	Porin	2.5	3.2873E-69	ChrII
VDA_003133	Glutamine synthetase type I	4.7	2.8174E-108	ChrI
VDA_000463	L-asparaginase	3.3	8.16042E-85	ChrII
VDA_003226	Glucosamine fructose-6-phosphate aminotransferase	3.5	3.71313E-35	ChrI
VDA_001560	Aspartate/tyrosine/aromatic aminotransferase	2.8	1.19637E-65	ChrI
A0J47_09785	Putative trypsin superfamily protein	4.3	4.6629E-132	ChrI
VDA_002568	Long-chain fatty acid transport protein	2.5	4.75483E-39	ChrI
VDA_000298	Arginine decarboxylase catabolic	2.6	7.62289E-83	ChrII
VDA_002194	Manganese-dependent inorganic pyrophosphatase	2.5	2.24757E-83	ChrI
VDA_003183	Oligopeptidase A	2.7	2.38104E-73	ChrI
VDA_003148	Vitamin uptake transporter	2.6	6.42707E-28	ChrI
*DNA synthesis and repair*				
VDA_002372	Ribonucleotide reductase of class Ia (aerobic) alpha subunit	3.5	1.03533E-70	ChrI
VDA_001894	Ribonucleotide reductase of class II (coenzyme B12-dependent)	3.1	6.75604E-90	ChrI
VDA_003108	Outer membrane vitamin B12 receptor BtuB	3.0	9.66178E-20	ChrI
VDA_001405	Uracil phosphoribosyltransferase	3.0	1.51133E-50	ChrI
*Translation*				
VDA_003390	LSU ribosomal protein L31p	2.7	1.98928E-09	ChrI
VDA_003099	Translation elongation factor Tu	2.6	3.07515E-36	ChrI
VDA_002952	SSU ribosomal protein S21p	2.6	1.9713E-24	ChrI
*Virulence and antimicrobial resistance*				
VDA_000110	Serum resistance protein Vep07-like	2.0	5.76819E-33	pPHDD1
VDA_000111	Transferrin receptor Vep20-like	3.0	5.67092E-76	pPHDD1
VDA_000113	OmpU	2.5	4.19858E-66	pPHDD1
VDA_000794	TonB-dependent siderophore receptor	2.8	5.7247E-111	ChrII
VDA_000157	TolC	2.8	2.115E-103	pPHDD1
VDA_000158	AcrA/MacA-like membrane fusion protein	3.2	7.9453E-112	pPHDD1
A0J47_18110	Unknown protein related to T6SS	2.8	1.0749E-24	pPHDD1
A0J47_18115	RNase toxin Ntox44	2.7	9.41221E-55	pPHDD1
A0J47_18120	Proline-alanine-alanine-arginine (PAAR) domain protein	2.9	1.3511E-60	pPHDD1
*Motility and chemotaxis*				
VDA_003029	Flagellar protein MotX	2.6	9.54565E-60	ChrI
VDA_002607	Flagellin protein FlaB	3.6	1.0743E-118	ChrI
VDA_002671	Flagellar motor rotation protein MotA	2.8	5.26275E-79	ChrI
VDA_002604	Flagellar biosynthesis protein FliS	2.7	6.88724E-93	ChrI
VDA_003044	Methyl-accepting chemotaxis protein	3.1	7.48432E-68	ChrI
VDA_001198	Methyl-accepting chemotaxis protein	2.7	1.10855E-85	ChrI
*Stress response and defence mechanisms*				
VDA_003059	Chaperonin complex GroEL-GroES	3.1	2.1437E-116	ChrI
VDA_003060	Chaperonin complex GroEL-GroES	3.5	1.517E-130	ChrI
VDA_002771	DnaK chaperonin	3.4	7.7384E-130	ChrI
VDA_001553	Peptidyl-prolyl cis-trans isomerase PpiD	2.5	3.30237E-65	ChrI
VDA_002523	HtpG chaperonin	2.8	3.0531E-107	ChrI
VDA_001325	ClpB chaperonin	2.5	3.25923E-77	ChrI
VDA_003124	Ribosome associated heat shock protein	3.0	1.98913E-71	ChrI
VDA_003529	Outer membrane stress sensor protease DegQ	3.3	1.13294E-15	ChrI
VDA_003386	ATP-dependent protease HslV	3.1	4.0286E-132	ChrI
VDA_001154	Peroxidase	4.9	6.6194E-111	ChrII
VDA_000806	Peroxidase	3.5	5.64336E-61	ChrII
VDA_000771	Iron-sulfur cluster assembly protein SufB	2.6	1.95494E-63	ChrII
*Transcriptional regulation and signalling*				
VDA_003227	DeoR family transcriptional regulator	3.3	1.4446E-35	ChrI
VDA_001088	XRE family regulator	2.6	7.86116E-25	ChrII
VDA_002825	Cyclic-di-GMP phosphodiesterase A	3.7	6.3346E-128	ChrI
*Cell wall/membrane/envelope biogenesis*				
VDA_003228	N-acetylglucosamine-1-phosphate uridyltransferase GlmU	2.7	4.3747E-86	ChrI
*Hypothetical proteins of unknown function*				
VDA_003431	Hypothetical protein	3.4	3.96176E-86	ChrI
VDA_000943	Hypothetical protein	2.6	3.53416E-24	ChrII
VDA_000598	Hypothetical protein	2.5	8.47329E-75	ChrII

#### Nutrient acquisition and metabolism

Genes encoding membrane proteins, nutrient transporters and porins were upregulated at 25°C (Table 1). The nucleoside permease NupC (VDA_001789) was the most upregulated gene at 25°C. A *V*. *cholerae nupC* deletion mutant was impaired for nucleoside acquisition leading to diminished fitness in nutrient-limited environments [44]. Growth at 25°C upregulated two ribonucleotide reductases belonging to class Ia and II, one of them, VDA_001894, is coenzyme B12-dependent. In accordance, the vitamin B12 receptor BtuB was also upregulated (Table 1). Upregulation of the uracyl phosphoribosyltransferase VDA_001405, an enzyme necessary for the synthesis of precursors of all pyrimidine nucleotides, was also observed. Among upregulated amino acid biosynthesis enzymes and aminotransferases was the glutamine synthetase type I, which has a central role in amino acid biosynthesis. The putative trypsin superfamily protein encoded by A0J47_09785, which has a possible function in peptide degradation, as well as a number of ribosomal and translation-related proteins were upregulated suggesting that growth at 25°C enhances protein synthesis and renovation.

*Pdd* degrades extracellular lipids, which may serve as carbon and energy sources [10, 11, 25]. An operon encoding an extracellular lipase (VDA_001610) and a fatty acid transporter FadL (VDA_001611) were 2-fold upregulated at 25°C, and so was the long-chain fatty acid transporter VDA_002568 (Table 1), suggesting that exogenous lipid degradation and uptake of fatty acids might constitute an advantage for *Pdd* fast replication at 25°C.

Iron acquisition plays a role in *Pdd* virulence for fish [45–47]. Our analysis unveiled upregulation at 25°C of a pPHDD1 plasmid-borne gene that encodes a putative transferrin binding protein (VDA_000111) (see below), and of VDA_000794 encoding a TonB-dependent siderophore receptor. The ligand(s) transported through VDA_000794 are unknown, but a recent study demonstrated that expression of this gene is enhanced in *Pdd* under iron-limitation conditions [48].

#### Motility and chemotaxis

Motility and tissue colonization constitute important factors in *Pdd* pathogenicity [39, 49]. Four flagellum-related genes were found among the 50 most upregulated genes (Table 1). Additional upregulated genes included flagellar hook protein FlgE (VDA_002616), flagellar motor rotation protein MotB (VDA_002670), flagellar hook-associated protein FlgK (VDA_002609) and flagellar basal-body rod modification protein FlgD (VDA_002617) (S3 Table). Previous studies have reported that *Pdd* can infect new hosts through seawater and that increased water temperature boosted infection by this route [49]. The upregulation of motility-related genes at 25°C supports these previous observations and surely calls for further studies along these lines.

Chemotaxis is initiated by membrane chemoreceptors dubbed methyl-accepting chemotaxis proteins, which bind ligands and transduce a signal cascade that modulates flagellum activity. Notably, two chemotaxis-related genes were found among the 50 most upregulated genes at 25°C and correspond to the methyl-accepting chemotaxis proteins VDA_003044 and VDA_001198 (Table 1). A recent study showed that mutants in chemotaxis genes are not only impaired in swimming motility in *Pdd* but also exhibit diminished production of the major virulence factor PhlyP and impaired adhesion to eukaryotic cells [50]. *Vibrio fischeri* and *V*. *anguillarum* mutants in chemotaxis functions also display a high reduction of virulence in fish and the inability to colonize host tissues [51, 52]. Collectively, these results suggest that growth of *Pdd* at 25°C could enhance chemotaxis and flagellum-dependent motility, contributing to access and adhesion to fish hosts and increasing chances of outbreaks in aquaculture farms during summer months.

#### Stress response

A number of chaperones, heat shock and stress-related proteins were listed among the 50-top DEGs (Table 1), suggesting that growth at 25°C constitutes a heat stress condition. Upregulation was also found for DnaJ chaperonin (VDA_002770), heat shock protein GrpE (VDA_002772) and heat-shock chaperonin VDA_003125 (S3 Table). Upregulated proteases included the outer membrane stress sensor protease DegQ and the ATP-dependent protease HslV, two peroxidases and the iron-sulfur cluster assembly protein SufB. SufB synthesizes Fe-S clusters that act as cofactors in cellular processes under conditions of iron starvation or oxidative stress in *E*. *coli* [53]. Altogether, it is conceivable that an increase in temperature might constitute a signal for *Pdd* to activate its molecular machinery against reactive oxygen species formation by host cells, therefore linking temperature rise with virulence for fish hosts.

#### Transcriptional regulators and intracellular signalling

A DeoR family transcriptional regulator and a XRE family regulator were listed within the top-50 DEGs. Although studies about these transcriptional regulators are scarce, in *Shigella flexneri* and *Salmonella typhi* DeoR regulators are important for virulence and for intracellular growth [54, 55]. Thus, these two genes may regulate processes related to *Pdd* pathogenicity and further investigation into these regulators is required. Cyclic-di-GMP is an intracellular second messenger involved in environmental signalling and regulates a number of phenotypes in bacteria. VDA_002825, encoding a cyclic-di-GMP phosphodiesterase A, was 3.67-fold up-regulated at 25°C. In *V*. *cholerae* cyclic-di-GMP regulates motility and biofilm formation [56, 57] and DNA repair [58], among other functions.

#### Secretion systems

The *Pdd* type II secretion system (T2SS) plays a major role in the secretion of the four cytotoxins Dly, PhlyP, PhlyC and PlpV [25, 26]. The complete cluster of *eps* (extracellular protein secretion) genes (VDA_003114-VDA_003123), encoding part of the T2SS machinery, was moderately upregulated at 25°C relative to 15°C (S3 Table), suggesting that the T2SS secretome serves important functions when this organism is growing at the temperature conditions that enhance outbreaks in aquaculture farms.

#### Potential virulence factors encoded within pPHDD1 plasmid are upregulated at 25°C

**Fig 4:**
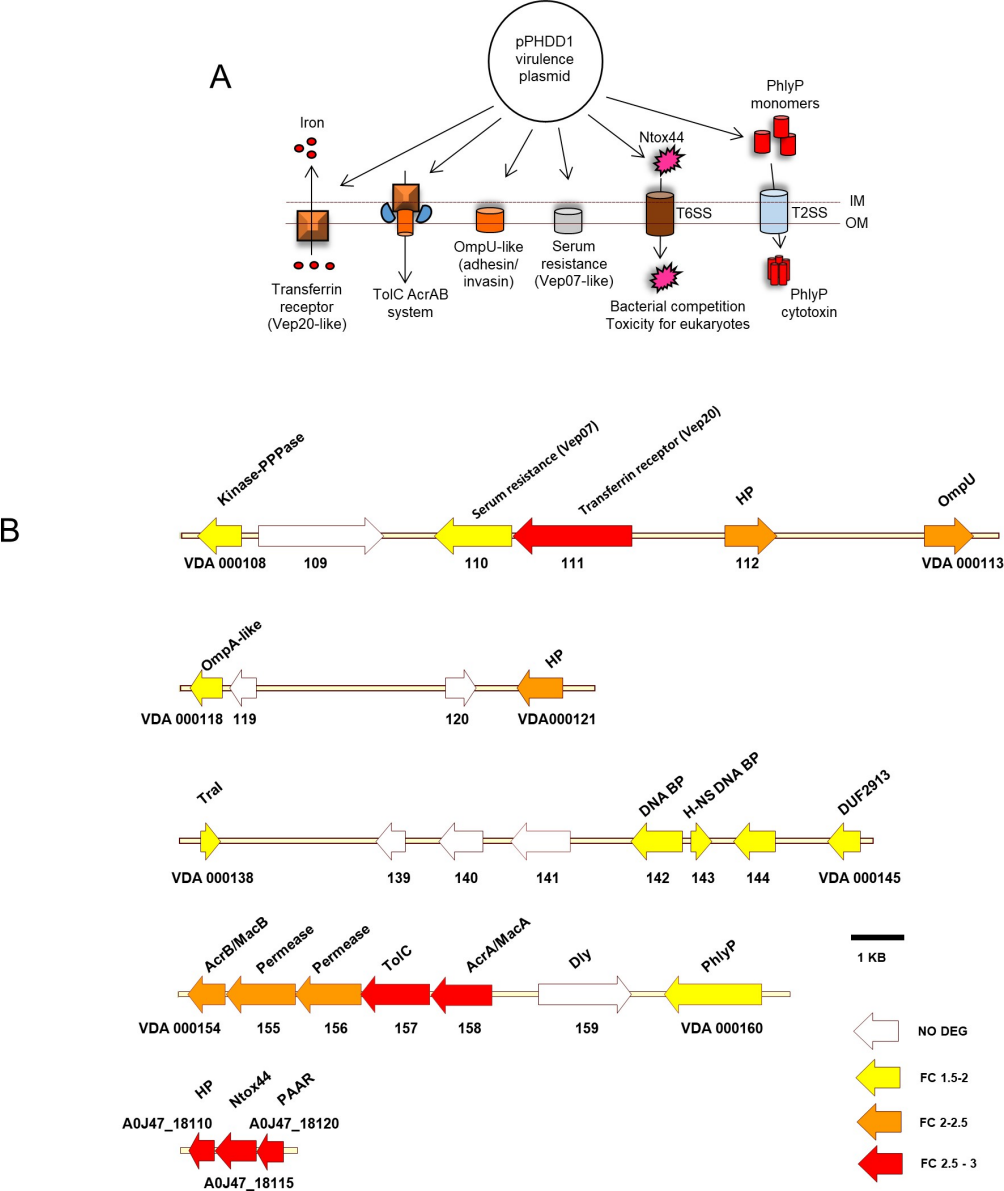
Virulence factors encoded within pPHDD1 plasmid are upregulated at 25°C. (A) pPHDD1 encodes a series of membrane-associated proteins and toxins with demonstrated and potential roles for virulence in fish. (B) plasmid genes upregulated at 25°C are mainly distributed along 5 plasmid regions. Numbers denote the VDA gene codes of the type strain CIP102761 (GenBank Acc. No. NZ_ADBS00000000.1). Genes with A0J47 labels correspond to genes unique to RM-71. Colour codes of genes (represented as arrows) denote each of the three ranges of FC values, whereas white arrows denote genes that are not differentially expressed (NO DEG).

A recent study reported that a double mutant of RM-71, with deletion of Dly and PhlyP-encoding genes, was still more virulent in a sea bass fish model than a naturally plasmidless strain [25], suggesting that pPHDD1 encodes additional yet uncharacterized virulence genes. The analysis of the differential gene expression profiles along pPHDD1 at the two temperatures brought to the forefront a collection of plasmid-encoded genes which have putative roles in virulence and that surely will deserve special attention in future studies (Fig 4, S5 Table).

A cluster of 5 upregulated genes (VDA_000154 to VDA_000158) encode the TolC protein and AcrAB, plus two additional proteins. TolC and AcrAB form a tripartite multidrug and toxin secretion efflux pump. Expression of *tolC* and *acrAB* in the fish pathogen *Yersinia ruckery* is increased at 28°C with respect to 18°C with a concomitant increased resistance to antibiotics and toxic substances such as acriflavine [59]. The role of this system in the biology of *Pdd* remains unknown. In addition to a possible role in efflux of toxic substances, we cannot rule out the possibility that this system participates in the secretion of virulence factors, as TolC is part of the type I secretion system that exports hemolysins and other virulence factors in several Gram negative pathogens [60].

VDA_000110 is 36% identical to *V*. *vulnificus* Vep07, an outer membrane lipoprotein that confers resistance to eel serum. Mutation of *vep07* caused the loss of virulence in eels in *V*. *vulnificus* biotype 2 [61]. Of note, *V*. *vulnificus* Vep07 is encoded within pVvbt2, a transferable virulence plasmid [62]. VDA_000113 is homologous to *Vibrio tasmaniensis* OmpU, a protein with an essential role in adhesion to and invasion of mollusc host cells [63]. OmpU homologues in other *Vibrio* species also play major roles in host-cell recognition and pathogenesis [64, 65]. Finally, the protein encoded by VDA_000111 shares 60% identity with the *V*. *vulnificus* plasmid-encoded Vep20 protein, a transferrin receptor with a major role for virulence in eels [66]. A previous study has demonstrated that *Pdd* strains can multiply in the presence of transferrin as the sole iron source [46]. Surely, the role of this gene deserves further investigation as it may play a main role in iron acquisition from host transferrin.

The three genes A0J47_18120, A0J47_18115 and A0J47_18110 are exclusive of RM-71 and have no known homologues in other pPHDD1-containing strains of *Pdd* studied so far. These genes are predicted to participate in defence against other bacteria which occupy the same ecological niches and compete for the same resources. A0J47_18120 is a proline-alanine-alanine-arginine (PAAR) domain-containing protein. Homologues of these proteins in *V*. *cholerae* are essential for secretion through the type VI secretion system (T6SS) and killing of target cells [67]. A0J47_18115 encodes a putative RNase toxin dubbed Ntox44 which is found in many bacterial groups and is predicted to be exported by the type II, type VI or type VII secretion systems [68].

### Growth at 25°C causes downregulation of functions related to cell envelope, metabolism and stress response

Albeit 15°C being far from the optimal growth temperature (25°C) of *Pdd* in laboratory conditions, it is conceivable that this bacterium lives in marine ecosystems in vast areas of the globe at temperatures below its optimum. Indeed, seawater temperatures during the summer of 1988 previous to the heat wave that caused a *Pdd* outbreak in a turbot farm in Galicia were around 18°C [10], and seawater temperatures in the same geographical area during the winter months are known to fluctuate between 13 and 15°C [69]. Growth at 25°C resulted in the downregulation of 614 genes that mapped to the genome of the type strain (S3 Table) and of 27 additional genes unique to strain RM-71 (S4 Table), whose changes in expression are denoted by a negative FC value. A list of the top-50 downregulated genes at 25°C, plus additional genes, is shown in Table 2 and reveals an important number of loci organized in operons.

**Table 2:** List of the top DEGs in *Pdd* with lower expression at 25°C than at 15°C. Note that downregulated expression at 25°C is denoted by negative FC values.

Gene ID	Product/Function	Fold Change	p-value	Location
*Cell wall/membrane/envelope biogenesis*				
VDA_001578	Stage V sporulation protein SpoVR family	-13.6	9.67E-189	ChrI
VDA_001762	Lysophosphatidic acid acyltransferase PlsC	-3.2	1.26692E-50	ChrI
VDA_002031	LrgB-family protein	-3.1	1.3247E-117	ChrI
*Various/unknown function*				
VDA_001579	Domain of unknown function, 444 superfamily	-28.2	0.0	ChrI
VDA_001580	PrkA-family serine protein kinase	-24.1	6.1873E-268	ChrI
VDA_001764	DedA superfamily member	-5.4	3.85608E-13	ChrI
*Glycine betaine transport*				
VDA_002013	Glycine betaine transporter	-7.8	1.07E-130	ChrI
*Nutrient transport and metabolism*				
VDA_001763	Putative cyanophicin synthetase	-7.1	2.98741E-37	ChrI
VDA_001723	Dipeptidase	-3.7	9.2245E-150	ChrI
VDA_000377	Metallopeptidase M24 family	-4.3	1.10747E-42	ChrII
VDA_001632	Oligopeptide ABC transporter OppA	-4.2	9.9638E-59	ChrI
VDA_001633	Oligopeptide ABC transporter OppB	-3.5	1.21575E-52	ChrI
VDA_001634	Oligopeptide ABC transporter OppC	-3.0	7.71823E-42	ChrI
VDA_001635	Oligopeptide ABC transporter OppD	-2.8	5.5165E-35	ChrI
VDA_001636	Oligopeptide ABC transporter OppF	-3.3	6.69827E-54	ChrI
VDA_001382	Methionine ABC transporter, substrate binding component	-3.3	4.52395E-44	ChrI
VDA_001251	Iron-molybdenum cluster-binding protein with NifB/NifX domain	-6.1	1.2384E-133	ChrI
VDA_001252	NADH:quinone oxidoreductase NqrM	-3.0	2.51284E-19	ChrI
VDA_002257	Alanine dehydrogenase	-4.3	7.76228E-76	ChrI
VDA_001997	Agmatinase	-3.9	9.89728E-95	ChrI
VDA_001605	Aspartate-semialdehyde dehydrogenase	-2.1	3.49578E-26	ChrI
VDA_002504	Citrate synthase	-3.5	5.08558E-45	ChrI
VDA_002724	Malate synthase	-3.1	8.95602E-40	ChrI
VDA_002723	Isocitrate lyase	-3.0	3.72706E-32	ChrI
VDA_000495	Putative hydrolase, alkyl/aryl sulfatase	-4.1	2.35121E-79	ChrII
VDA_002425	Acetoacetyl-CoA-reductase	-3.7	4.67185E-25	ChrI
VDA_002349	Alpha acetolactate decarboxylase	-3.3	3.9868E-50	ChrI
VDA_001166	Alpha-1,2-mannosidase	-3.2	1.30795E-38	ChrII
*Stress response and host defence*				
VDA_003169	Cold-shock protein	-4.6	8.21889E-58	ChrI
VDA_000629	RpoS	-3.5	5.16984E-57	ChrII
VDA_001327	Glutamate decarboxylase	-3.3	9.17337E-45	ChrI
VDA_001328	Glutaminase	-3.4	2.2451E-42	ChrI
VDA_001329	Glutamate/GABA antiporter	-3.0	7.67865E-16	ChrI
VDA_001116	Multidrug resistance efflux pump	-3.5	6.15611E-72	ChrII
VDA_000570	Methionine sulfoxide reductase MsrQ	-3.1	9.25296E-34	ChrII
VDA_000571	Methionine sulfoxide reductase MsrP	-4.6	1.28296E-51	ChrII
VDA_001099	Cu-Zn Superoxide dismutase	-3.1	1.9361E-122	ChrII
*Virulence factors*				
VDA_002242	Phospholipase PlpV	-1.8	2.02363E-17	ChrI
*Hypothetical proteins of unknown function*				
VDA_000632	Hypothetical protein	-6.2	8.1218E-133	ChrII
VDA_001326	Hypothetical protein	-3.4	2.00678E-63	ChrI
VDA_000814	Hypothetical protein	-3.8	7.28892E-46	ChrII
VDA_000647	Hypothetical protein	-3.6	2.93637E-36	ChrII
VDA_001949	Hypothetical protein	-3.5	1.82412E-53	ChrI
VDA_001746	Hypothetical protein	-3.4	1.43464E-49	ChrI
VDA_002346	Conserved hypothetical membrane protein	-3.3	1.30302E-29	ChrI
VDA_001892	Putative transporter	-3.1	4.50201E-55	ChrI
VDA_001660	Hypothetical protein	-3.0	2.70017E-34	ChrI
VDA_003210	Hypothetical protein	-3.0	4.74045E-89	ChrI
VDA_000496	Hypothetical protein	-3.0	7.01092E-40	ChrII
VDA_003208	Hypothetical protein	-3.0	8.59014E-50	ChrI
VDA_000405	Hypothetical protein	-3.0	9.44929E-53	ChrII
VDA_002379	Hypothetical protein	-3.0	4.8787E-117	ChrI
VDA_000439	Hypothetical protein	-3.0	1.36579E-08	ChrII

The most important change is experienced by a putative operon constituted of VDA_001578, VDA_001579 and VDA_001580 (Table 2). VDA_001579 is the most differentially expressed gene in the whole transcriptome of this pathogen in this study, and has no known homologues with a demonstrated function so far. VDA_001580 is a predicted serine protein kinase PrkA, a family of proteins involved in cell wall homeostasis [70], saline stress, motility [71] and virulence [72]. VDA_001578 is predicted to be a member of the Stage V sporulation protein SpoVR family. SpoVR confers resistance to *Bacillus subtilis* spores and it has been hypothesized that homologues in other species might play a role in peptidoglycan synthesis regulation [73]. Also related to the cell wall is VDA_002031, which encodes a LrgB-family protein, a group of enzymes responsible for modulation of murein hydrolase activity [74]. VDA_001762 encodes lysophosphatidic acid acyltransferase PlsC, an integral membrane protein involved in phospholipid biosynthesis [75]. Adjustment of membrane composition is a conserved strategy that bacteria use to face variations in environmental parameters [76, 77]. The importance of the cell envelope in *Pdd* acclimatization to temperature changes surely will deserve further investigation.

Growth at 25°C downregulated the expression of peptidases, membrane transporters and metabolic enzymes (Table 2). Of note is the downregulation of the oligopeptide permease system *oppABCDF* whose main function is predicted to be nutritional [78]. Interestingly, the expression of *oppABCDF* in *Vibrio alginolyticus* was found to be sensitive to temperature [79]. The role of the *opp* operon in *Pdd* physiology has not been studied so far and further evaluation of these genes is necessary to assess their possible contribution to bacterial fitness at low temperatures.

VDA_001251 contains a NifB/NifX domain for synthesis of iron-molybdenum cofactors. These cofactors bind the active site of dinitrogenase enzyme which participates in nitrogen fixation [80]. Although nitrogen fixation has not been studied in *Pdd*, some members of the *Vibrionaceae* family have this ability [81]. Enzymes of amino acid metabolism were also downregulated at 25°C. The upregulation of specific amino acid biosynthetic pathways at both temperatures might be in part attributable to the need to provide optimal amino acid ratios for the different types of proteins produced at each temperature.

Genes of the Krebs cycle were downregulated at 25°C: citrate synthase, malate synthase and isocitrate lyase. In addition to its role in the cell bioenergetics, the Krebs cycle is connected to iron acquisition in *Pdd* where endogenous citrate is used as iron scavenger [45, 82]. Thus, the use of ferric citrate as an iron source might be favoured in *Pdd* at low temperatures.

Biosynthesis and uptake of betaine glycine constitutes an adaptation for growth at low temperatures and is part of the cold-stress response of the marine fish pathogen *Vibrio anguillarum* [83]. Of note, a glycine betaine transporter showed a strong downregulation at 25°C in our study (Table 2). Only one gene among the main 50 downregulated genes at 25°C encoded a cold shock protein (VDA_003169). In contrast, some mesophilic bacteria such as *V*. *cholerae* [84] and *V*. *parahaemolyticus* [85] overexpress cold shock proteins following shifts to low temperatures close to 15°C. The alternative sigma factor RpoS (VDA_000629) is downregulated at 25°C. RpoS is a major regulator of the general stress response pathway in bacteria [86]. Important traits regulated by RpoS include virulence and colonization in *V*. *cholerae* and *V*. *parahaemolyticus* [87, 88]. A three-gene operon encoding a glutamate decarboxylase, a glutaminase and a glutamate/GABA antiporter is potentially involved in acid resistance [89]. The biological roles of this system in *Pdd* are unknown, but amino acid decarboxylation has been reported in other species of the *Vibrionaceae* family as a strategy for acid tolerance [90]. An operon which encode the methionine sulfoxide reductase system MsrPQ was downregulated in *Pdd* at 25°C. The MsrPQ systems participate in the repair of oxidative damage [91].

### Growth at 25°C does not upregulate expression of the major cytotoxins of *Pdd*: Dly cytotoxin is within the 10 most expressed genes at 15°C

Considering their importance in the pathogenicity of *Pdd* for fish, expression of the cytotoxins Dly, PhlyP, PhlyC and PlpV would be expected to be upregulated at 25°C. Unexpectedly, expression of damselysin (Dly) (VDA_000159), PhlyP (VDA_000160) and PhlyC (VDA_002420) did not experience significant expression changes in growth at 25°C compared to 15°C (S3 Table), and PlpV (VDA_002242) was slightly downregulated at 25°C (Table 2). This clearly indicates that levels of cytotoxin expression at 15°C do not represent a limiting step that would prevent disease outbreaks in fish from occurring at low temperatures. These observations support that, as suggested above, the major influence of increased seawater temperatures in the onset of *Pdd* outbreaks in fish farms may be connected to the upregulation of other cellular processes (higher division rate, motility, chemotaxis, other plasmid-encoded putative virulence factors, etc) and not to a differential production of the four major cytotoxins.

This observation prompted us to analyze the RNAseq data to identify which are the most expressed genes in the genome of *Pdd* at each temperature of the study. Transcript abundance was quantified as Fragments Per Kilobase of transcript per Million mapped reads (FPKM), a method that allows the comparison of transcripts abundance among samples and conditions (S6 Table). Notably, the *dly* gene was the ninth most expressed gene at 15°C (Table 3) with transcript abundance levels similar to genes of ribosomal proteins, which are among the most actively transcribed genes in fast-growing prokaryotic cells [92]. Two cold shock proteins and the NAD-dependent glyceraldehyde-3-phosphate dehydrogenase were included within the 10 most expressed genes at 15°C. The 10 most expressed genes at 25°C all corresponded to ribosomal protein genes (Table 4). LSU ribosomal protein L24p (L26e) (VDA_003450) was the most expressed gene under both conditions.

**Table 3:** List of most expressed genes at 15°C and their corresponding FPKM values. Damselysin (Dly) toxin is highlighted in bold. FPKM values shown correspond to mean values of the three biological replicates.

Locus tag	Protein	FPKM
VDA_003450	LSU ribosomal protein L24p (L26e)	14782
VDA_000346	Putative cold shock-like protein	14687
VDA_003244	LSU ribosomal protein L34p	14467
VDA_003169	Cold shock protein	12798
VDA_001583	NAD-dependent glyceraldehyde-3-phosphate dehydrogenase	12190
VDA_003449	LSU ribosomal protein L14p (L23e)	11923
VDA_003447	LSU ribosomal protein L29p (L35e)	11833
VDA_003446	LSU ribosomal protein L16p (L10e)	11690
VDA_000159	**Damselysin toxin (Dly)**	10938
VDA_003456	SSU ribosomal protein S5p (S2e)	10255

**Table 4:** List of most expressed genes at 25°C and their corresponding FPKM values. FPKM values shown correspond to mean values of the three biological replicates.

Locus tag	Protein	FPKM
VDA_003450	LSU ribosomal protein L24p (L26e)	16486
VDA_003447	LSU ribosomal protein L29p (L35e)	15622
VDA_003093	LSU ribosomal protein L7/L12 (L23e)	15513
VDA_003446	LSU ribosomal protein L16p (L10e)	14870
VDA_003461	SSU ribosomal protein S13p (S18e)	13609
VDA_003444	LSU ribosomal protein L22p (L17e)	13577
VDA_003449	LSU ribosomal protein L14p (L23e)	13557
VDA_003456	SSU ribosomal protein S5p (S2e)	13188
VDA_003244	LSU ribosomal protein L34p	12838
VDA_003463	SSU ribosomal protein S4p (S9e)	12813

**Fig 5:**
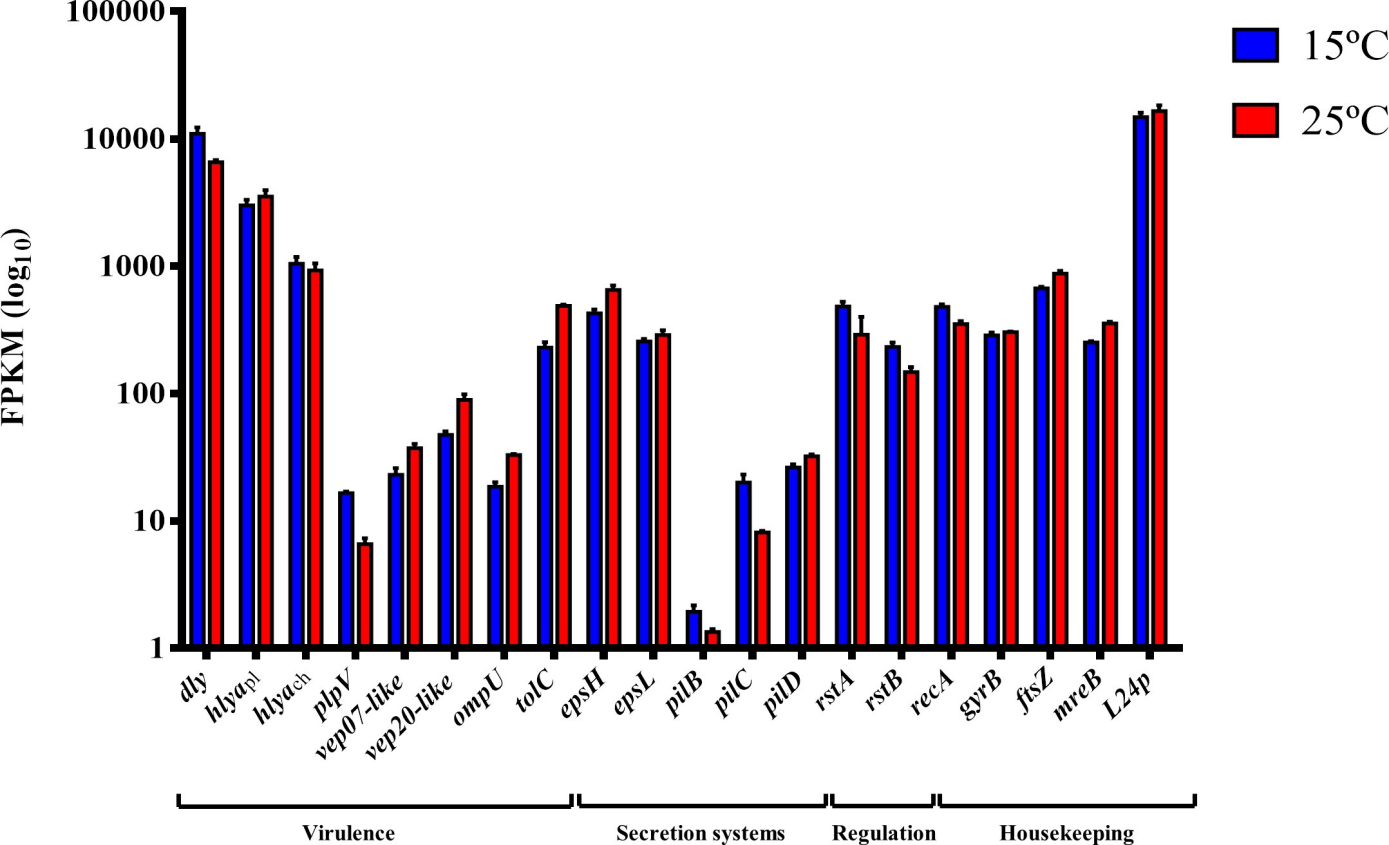
Damselysin toxin gene *dly* is one of the most highly expressed genes in *Pdd*. FPKM (Fragments Per Kilobase of transcript per Million mapped reads) values at the two assayed temperatures (S6 Table) were obtained for a selection of virulence and regulatory genes, secretion system genes and housekeeping genes including the top-expressed gene encoding ribosomal protein L24p, and compared using a logarithmic scale. Vertical error bars represent standard deviation of biological triplicates.

To illustrate the dominance of damselysin toxin transcripts, the FPKM values of *dly* and other virulence-related genes, as well as a selection of genes related with secretion systems and housekeeping cellular functions were compared (Fig 5). Although far from the top 10 most expressed genes, the FPKM values of the mRNA levels for the two pore-forming toxins PhlyP and PhlyC were also higher than those of housekeeping genes as *gyrB*, *recA*, *mreB* and *ftsZ*. Dly was the most highly expressed virulence factor at the two temperatures of the study, being particularly the case at 15° C, and its transcript levels were almost 3 orders of magnitude higher than those of the PlpV phospholipase, which is considered to only have a minor contribution to virulence and cell toxicity [25]. The plasmid-encoded putative virulence factors OmpU, Vep07 and Vep20 showed transcript abundance levels largely inferior to Dly cytotoxin, again reinforcing the dominance of Dly as the top-expressed virulence factor in *Pdd*. This observation is in agreement with early studies which described highly virulent *Pdd* strains are producers of “large amounts of a cytolytic toxin in vitro” [93] and reinforces the major role of pPHDD1 as a virulence plasmid that contributed to the evolution of highly hemolytic and highly virulent lineages of *Pdd*.

## Conclusions

**Fig 6:**
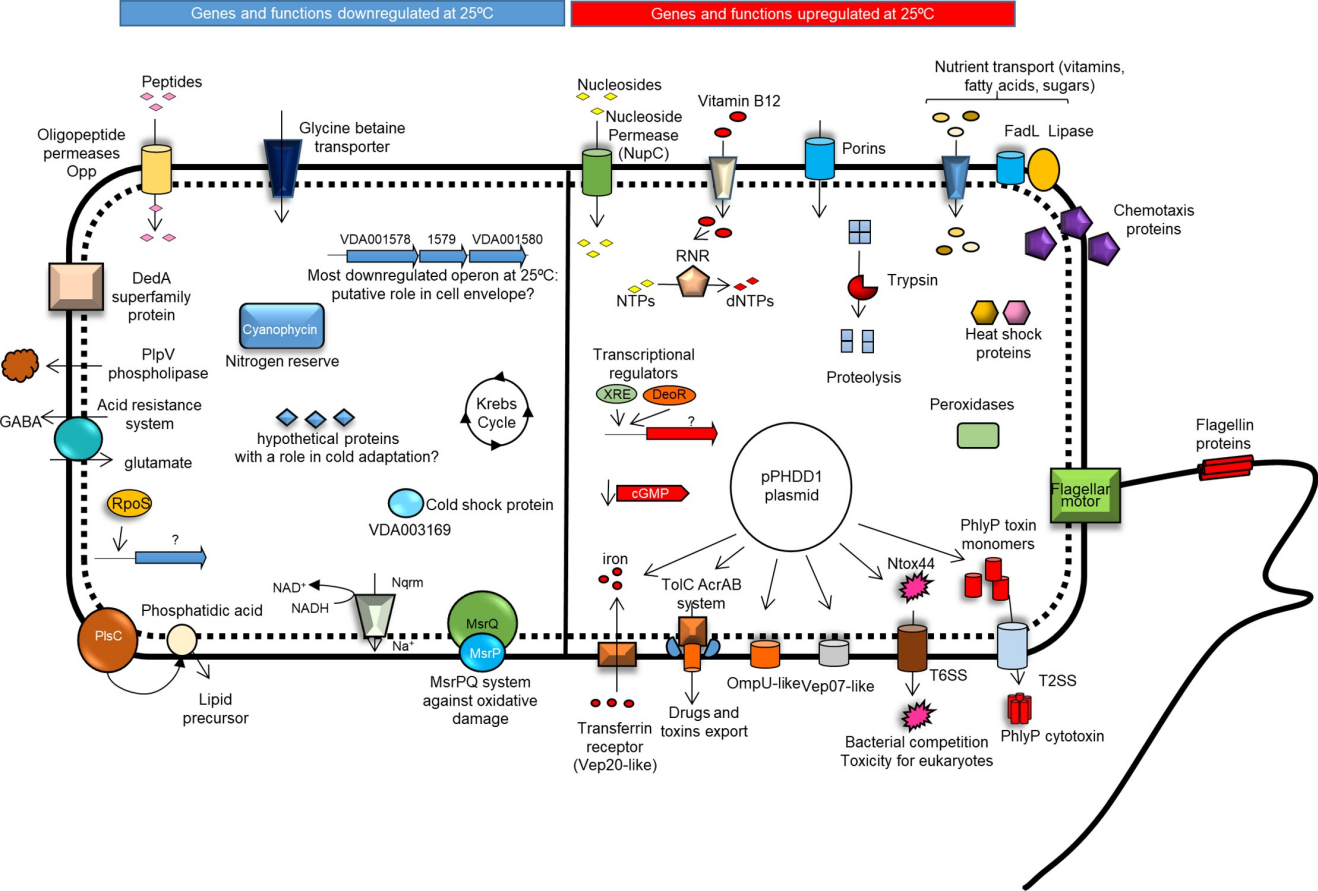
**Diagrammatic summary of genes upregulated (right side) and downregulated (left side) at 25°, relative to 15°C, in *Pdd*.** Growth at 25°C upregulated motility and chemotaxis-related functions, as well as nutrient uptake and utilization genes. Potential virulence factors encoded within pPHDD1 plasmid were also upregulated at 25°C. Genes with lower expression at 25°C include a number of gene operons of yet unknown function, functions related to cell envelope, *rpoS*, and specific amino acid biosynthesis routes, among others. This diagram has been constructed based on transcriptomic data, and further studies are needed to establish the proposed model.

Regulation of expression of virulence factors in response to temperature has been widely studied in pathogenic bacteria infecting homeotherms [94–96]. Meanwhile, much less is known about the temperature-dependent regulation of virulence factors in fish bacterial pathogens [59, 97]. The aim of the current study was to investigate which genes are differentially regulated in *Pdd* in a heat stress condition (25°C), relative to a colder condition (15°C), considering that the majority of fish farm outbreaks occur during warm summer months at temperatures close to 25°C. Fig 6 features a diagram of differentially expressed functions that are upregulated (right panel) or downregulated (left panel) at 25°C.

Growth at 25°C resulted in the upregulation of motility- and chemotaxis-related functions, as well as nutrient uptake and utilization genes. Notably, potential virulence factors encoded within pPHDD1 related to iron acquisition (transferrin receptor), adhesion (OmpU), serum resistance (Vep07-like) and defence against competitors, were also upregulated at 25°C (Fig 6). Overall, this study unveils a large set of previously overlooked genetic networks in *Pdd* and points at a number of cell functions related to virulence and acclimatization to changes in the environment.

Notably, damselysin toxin was one of the most highly expressed genes in the cell at the two assayed temperatures, with expression levels comparable to the most expressed genes encoding ribosomal proteins. This finding highlights the importance of this phospholipase D for the bacterium and suggests that this toxin might fulfil other biological roles in addition to virulence. To the best of our knowledge, this study represents the first transcriptome-based analysis of *Pdd*, and it has allowed us to identify a large set of gene functions that surely will constitute a foundation for future studies. Currently we are in the process of investigating the role of the top-differentially expressed genes identified in this study in the pathobiology of *Pdd*.

## Supporting information

S1 Table OD_600_ data for each biological replicate at 15°C and 25°C used to generate growth curves depicted in Fig 1. (XLSX)

S2 Table Reads mapping for each biological replicate. (XLSX)

S3 Table List of Differentially Expressed Genes (DEGs) at 25°C vs 15°C mapped to type strain genome (CIP102761). (XLSX)

S4 Table List of Differentially Expressed Genes (DEGs) at 25°C vs 15°C mapped to RM-71 genome. (XLSX)

S5 Table List of Differentially Expressed Genes (DEGs) within virulence plasmid pPHDD1. (XLSX)

S6 Table FPKM values for each biological replicate at 15°C and 25°C. (XLSX)
